# Novel correction procedure for compensating thermal contraction errors in the measurement of the magnetic field of superconducting undulator coils in a liquid helium cryostat

**DOI:** 10.1107/S1600577524000808

**Published:** 2024-02-22

**Authors:** Barbara Marchetti, Johann Baader, Sara Casalbuoni, Grigory Yakopov, Mikhail Yakopov

**Affiliations:** a European X-ray Free-Electron Laser, Holzkoppel 4, 22869 Schenefeld, Germany; Paul Scherrer Institute, Switzerland

**Keywords:** superconducting undulators, FEL, magnetic field measurement, cryostat, data analysis

## Abstract

Superconducting undulators (SCUs) can offer a much higher on-axis undulator field than state-of-the-art cryogenic permanent-magnet undulators with the same period and vacuum gap. The development of shorter-period and high-field SCUs would allow the free-electron laser and synchrotron radiation source community to reduce both the length of undulators and the dimensions of the accelerator. Magnetic measurements are essential for characterizing the magnetic field quality of undulators for operation in a modern light source. Hall probe scanning is so far the most mature technique for local field characterization of undulators. This article focuses on the systematic error caused by thermal contraction that influences Hall probe measurements carried out in a liquid helium cryostat. A novel procedure, based on the redundant measurement of the magnetic field using multiple Hall probes at known relative distance, is introduced for the correction of such systematic error.

## Abstract 2

A novel correction procedure, for compensating thermal contraction errors in the measurement of the magnetic field of superconducting undulator coils in a liquid helium cryostat by using measurements made with multiple Hall probes placed at fixed distance, is proposed. The effectiveness and limitations of the correction procedure are validated by numerical simulations.

## 1. Introduction

In recent years, superconducting undulators (SCUs) have been successfully employed at synchrotron radiation sources (Casalbuoni *et al.*, 2018 ▸; Ivanyushenkov *et al.*, 2018 ▸). SCUs can offer a much higher on-axis undulator field than state-of-the-art cryogenic permanent-magnet undulators with the same period and vacuum gap (Elleaume *et al.*, 2000 ▸; Bahrdt & Gluskin, 2018 ▸). Similarly to what has been demonstrated for synchrotrons, the use of SCUs can potentially improve the performance and flexibility of advanced free-electron lasers (FELs). In particular, by working at short undulator periods, superconducting magnet technology would allow higher photon energies to be reached while keeping a wide range of tunability of the setpoint. That is why the implementation of SCUs is being considered by several FEL projects world-wide (SLAC, 2020 ▸; Tang *et al.*, 2020 ▸). Research and development of superconducting undulator coils requires building up test stands for characterization of the coils (Mashkina *et al.*, 2008 ▸; Harder *et al.*, 2005 ▸; Grau *et al.*, 2016 ▸; Kasa *et al.*, 2019 ▸). Increasing stringent mechanical requirements on the coils’ precision calls for development of high-precision diagnostics for their characterization (Zhang & Calvi, 2022 ▸). Casalbuoni *et al.* (2023 ▸) discussed how the European XFEL facility benefits from the ongoing research and development program on SCUs for possible future upgrade of the accelerator towards producing harder X-rays and improving the tunability of the beamline. The extension of the energy range of the radiation towards higher values would fully exploit the high electron energy beam capability of the accelerator (Decking *et al.*, 2020 ▸). Moreover, this development can be considered complementary to studies on the upgrade of the XFEL linac for continuous-wave operation (Brinkmann *et al.*, 2014 ▸).

A project for the construction of a SCU afterburner at the SASE2 beamline at European XFEL has been recently proposed. Casalbuoni *et al.* (2021 ▸, 2022 ▸) presented the design parameters of the coils of a prototype module, named S-PRESSO (Superconducting undulator PRE-SerieS mOdule). The required mechanical accuracy for S-PRESSO is reported in Table 2 of Casalbuoni *et al.* (2023 ▸). The values have been set considering the following specifications for the undulator parameter: |Δ*K*/*K*|_RMS_ < 0.0015 and |Δ*K*/*K*| < 0.006. FEL simulations show that the same requirements on the mechanical accuracy are also well compatible with a complete SCU line with 15 mm period length. Such mechanical accuracy reduces the mean FEL power at the saturation length by less than 5% (Marchetti *et al.*, 2022*a* ▸). In order to resolve the given specification in a coil having a peak magnetic field equal to 1.8 T, it is necessary to measure the variations of the magnetic field profile Δ*B*/*B* at the 10^−3^ level, *i.e.* the resolution of the absolute value of the magnetic field must be better than 1.8 mT. Moreover, an accuracy and resolution of the Hall probe position of roughly 1 µm is desired to be able to resolve errors in the manufacturing of the pole/groove width of the coil of the order of 10 µm. In order to perform the quality assurance of the SCU coils for S-PRESSO and future SCU modules, two test stands, named SUNDAE1 (Superconducting UNDulAtor Experiment 1) (Marchetti *et al.*, 2022*b* ▸) and SUNDAE2 (Baader *et al.*, 2022 ▸), are presently under construction at the DESY campus. SUNDAE1 consists of a vertical cryostat hosting a liquid helium or super-fluid helium bath in which superconducting undulator coils for future application at European XFEL can be characterized prior to installation in their final cryostat.

Fulfilling the requirement regarding the accuracy of the positioning of the Hall probe used for the magnetic field scan is not trivial. It requires investigation and compensation of the systematic errors affecting the measurement. This article considers the setup of a general vertical cryostat and focuses on the errors that are produced by the contraction of the rod holding the Hall probe.

Our article is organized as follows. In Section 2 the geometry of the model used to simulate the thermal contraction of the rod during the Hall probe scan will be introduced and the correction procedure for systematic error generated during the magnetic field measurement will be presented. In Section 3 the validity of the proposed method will be benchmarked with simulations of magnetic field measurements under different experimental conditions. The study cases have been grouped based on the observed behaviour of the contraction of the rod during the scan of the Hall probe. We start considering physical situations in which the contraction of the rod varies linearly with the position of the Hall probe (Subsection 3.1) and we continue showing physical cases in which the contraction of the rod varies non-linearly during the scan (Subsection 3.2). In most of the practical cases it will be possible to compensate this error by measuring the field profile using only two Hall probes. The implications of this study for SUNDAE1 will be discussed in Section 4 and more general conclusions will be summarized in Section 5.

## 2. Method

### 2.1. Modelling the contraction of the rod during the scan of the Hall probe position

State-of-the-art SCUs used in accelerators have a horizontal cryostat that allows the alignment of undulator segments along the beam direction. Such cryostats are typically cooled down using conduction cooling with Gifford–McMahon cryocoolers (Casalbuoni *et al.*, 2018 ▸) or by a thermosyphon principle including a tank of liquid helium (Ivanyushenkov *et al.*, 2018 ▸). Opposite to this, the magnetic field characterization is often carried out in vertical cryostats hosting the magnet in a liquid helium bath.

**Figure 1:**
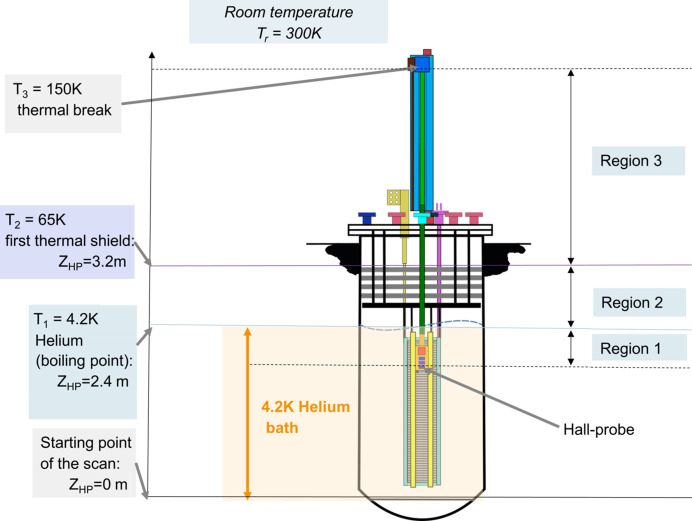
Sketch of the geometry used for modelling the magnetic field measurement. The baseline figure represents the cryostat and it is modified from Marchetti *et al.* (2022*b* ▸). On the axis on the left, the temperature values in the cryostat for different effective positions of the Hall probe *z*_HP_ are shown. The portion of the cryostat filled with liquid helium is highlighted in yellow. On the right side of the sketch the three regions defined for the model of the rod contraction are shown, for the specific depicted position of the Hall probe. Region 1 represents the part of the rod that is immersed in the liquid helium. Region 2 represents the part of the rod that is located between the liquid helium surface and the first thermal shield of the lid. Region 3 represents the part of the rod that is located between the first thermal shield and the thermal break at the upper flange of the linear motion system.

Fig. 1 ▸ shows a sketch of the general measurement setup under consideration. The magnet is hosted in a liquid helium bath and the measurement of the magnetic field is made by scanning the position of one Hall probe. The Hall probe is hosted in a sledge connected to a rod and to a linear motion system. The operation of the magnet increases the heat load in the cryostat. In vertical cryostats the helium level can be guaranteed by manual refill (Mashkina *et al.*, 2008 ▸), interface with a dewar (Bertucci *et al.*, 2019 ▸) or a cryogenic plant (Schaffran *et al.*, 2014 ▸; Perin *et al.*, 2010 ▸). If the helium level is refilled at chosen points of the measurement, *e.g.* before the start or at the end of it, the helium evaporation during the measurement is not compensated. In modern setups it is possible to monitor the level of the helium surface during the measurement and to feed it back to a Joule Thomson valve controlling the helium flow (Putselyk, 2020 ▸; Böckmann *et al.*, 2015 ▸; Pei *et al.*, 2014 ▸). This mechanism keeps the helium level constant, typically down to few cm level variation.

**Figure 2:**
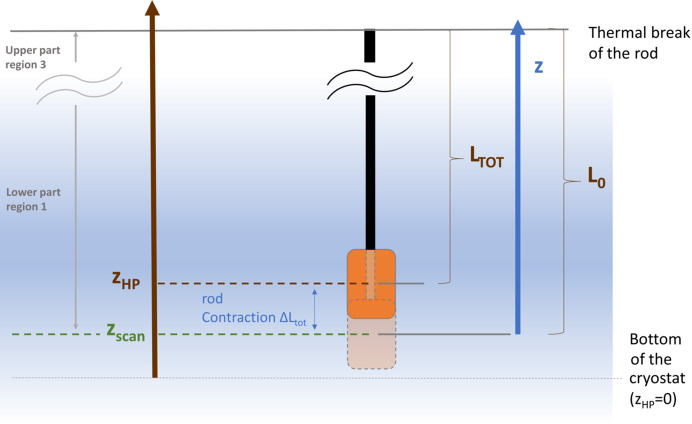
Definition of the longitudinal variables used in the numerical model that simulates the contraction of the rod. *z*_scan_ and *z*_HP_ are defined in the laboratory system and represent the longitudinal coordinate measured by the encoder of the linear motion system and the real position of the Hall probe, respectively. The *z*-axis is defined in the reference system of the rod before that the rod contraction is calculated. The *z* variable will be used to express the temperature profile along the rod. On the left side of the plot the corresponding regions of the cryostat as defined in Fig. 1 ▸ are marked. The double waves indicate a cut in sketch.

The rod supporting the sledge and Hall probe, shown in Fig. 1 ▸, is only partially immersed in liquid helium. The fraction of the rod that is immersed in liquid helium depends on the longitudinal location of the Hall probe monitored by the encoder of the linear motion system, *z*_scan_. As depicted in Fig. 2 ▸, the elongation or contraction of the rod by a few mm during the scan, due to the change of temperature gradient along it, influences the relative position of the Hall probe with respect to the SCU.

In this study we aim to reproduce the systematic error caused by the expansion/contraction of the rod during the scan of the magnetic field profile. For this reason, several routines in Matlab (MathWorks, 2023 ▸), that simulate the scan of the Hall probe position along the longitudinal coordinate, have been developed. At each scan point the value of the position of the Hall probe, *z*_scan_, read by the linear motion system’s encoder, which is assumed to be calibrated at room temperature, is stored. The actual position of the Hall probe, *z*_HP_, considers the rod’s effects of contraction and expansion (see Fig. 2 ▸).

Several models have been employed for the estimation of the dynamics of the contraction of the rod. A common feature is that the rod is divided into three regions as depicted in Fig. 1 ▸. The three regions are defined as follows:

(i) Region 1 has a variable length and represents the part of the rod that is immersed in liquid helium. At the beginning of the position scan, the Hall probe is assumed to be located at the bottom of the cryostat, where *z*_HP_ = 0; therefore the length of region 1 decreases while *z*_HP_ increases.

(ii) Region 2 represents the part of the rod that is temporarily located between the liquid helium surface and the first thermal shield of the lid. In our study the scan range has been limited to 2 m, therefore the Hall probe never leaves the liquid helium surface and region 2 has a fixed length. The rod travelling in region 2 experiences a temperature gradient between 4.2 K (on the liquid helium extreme) and 65 K (at the position of the first thermal shield).

(iii) Region 3 represents the part of the rod that is temporarily located between the first thermal shield and the thermal break at the upper flange of the linear motion system. This region has a variable length that increases during the scan, since the Hall probe and the connected rod are moved towards the outside of the liquid helium bath. The rod in this region experiences a temperature gradient between 65 K (at the position of the first thermal shield) and about 150 K (at the position of the thermal break of the upper flange of the linear motion system).

The calibration of the encoder controlling the motion of the Hall probe refers to room temperature conditions. If the rod is brought to a constant temperature *T*_1_, it will contract by 

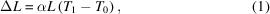

where α is the thermal expansion coefficient of the material constituting the rod, *T*_0_ = 300 K is the room temperature and *T*_1_ < *T*_0_. In our case, the rod will not experience a constant temperature but a temperature gradient. In the study it has been assumed that the scan of the position of the Hall probe is slow enough (*i.e.* several hours long) to guarantee that the rod reaches thermal balance with the environment (steady state condition) at each scanned point. We also assume to know the value of the rod temperature *T*(*z*) in the cryostat along the *z*-axis, defined in Fig. 2 ▸. Finally, α has been considered constant along the temperature range of the scan. The latter condition is not true since it is expected for α to vary by roughly one order of magnitude in the range of temperature 4–150 K (Hildnert, 1943 ▸; Bartosik *et al.*, 2017 ▸) but this assumption simplifies the model significantly. The implications of this premise will be better commented in Section 5.

Under such conditions, we can calculate the total contraction of the rod by 

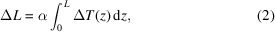

where Δ*T*(*z*) = *T*(*z*) − *T*_0_.

In our numerical model the rod is divided into the three regions defined in Fig. 1 ▸. Afterwards, the calculation of the contraction/expansion of the rod in one region at a time takes place, starting from the top, *i.e.* from region 3. It is important to mention that, since the rod is physically anchored to the thermal shield at the upper edge of region 3, the length of regions 3 and 2 at each *z*_scan_ position can simply be determined by looking at the geometry of the scan. Opposed to this, the calculation of the length of region 1 requires taking into account also the contraction of the rod in regions 2 and 3, since the edge of the rod where the Hall probe is located is free to move.

The actual position of the Hall probe, *z*_HP_, is given by *z*_HP_ = *z*_scan_ + Δ*L*_tot_, *i.e.* by adding to the reference value given by the encoder, *z*_scan_, the total contraction/expansion value of the rod. Note that, in the notation of equation (1), Δ*L*_tot_ can have a negative sign.

In our study we have considered a constant thermal expansion coefficient α = 8.6 × 10^−6^ K^−1^ as for the titanium alloy that we plan to use to build the rod in SUNDAE1. The value available in the literature for the chosen alloy is given in the range of temperatures 293.15–373.15 K (MatWeb, 2023 ▸).

Two shapes for the temperature distribution *T*(*z*) along the rod have been implemented:

(i) *T* ≃ *z*, which corresponds to the steady-state case in which there is no internal heat deposition, for a material having constant thermal conductivity in the temperature range of interest (Parma, 2014 ▸).

(ii) *T* ≃ exp(−*az*), which is an example of more general temperature profile used to match experimental data collected in steady-state conditions (Glasgow, 2009 ▸).

The following sections are devoted to the characterization of an undulator with a period length λ_u_ = 18 mm. The focus is on the systematic error for a magnetic scan covering a range of number of periods between 50 and 111, which corresponds to a scan length between 0.9 m and 2 m. This article does not address the measurement of the end fields of the coils.

### 2.2. Derivation of the correction procedure for systematic errors in the measurement of the period length

This section is centred on the introduction of a novel method for the correction of the systematic errors that arise in the measurement of the magnetic field of the coils. This method relies on the redundant measurement of the magnetic field profile by using two or more Hall probes installed on the sledge and placed at fixed known relative distance from each other. For the method to work, it is essential that the distance in cold conditions between the Hall probes remains constant, *i.e.* the sledge must not exit the liquid helium bath.

The goal is to show that the error in the measured value of the period can be compensated by determining a correction factor that accounts for the contraction of the rod when measuring each undulator period. It has been already defined,

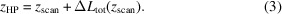

Δ*z*_HP_ can be expressed as

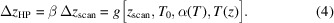

The scanning range of the Hall probe can be divided into small intervals. In each one of the given intervals the general function *g*[*z*_scan_, *T*_0_, α(*T*), *T*(*z*)] can be approximated using a Taylor expansion as

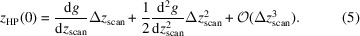

First of all let us consider what happens if only the first order of the expansion is taken into account. For each small interval of our scan it is possible to write

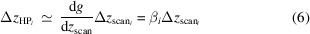

where β_*i*_ is calculated in a specific interval *i* of the scan which has the length of one period of the undulator to be characterized.

**Figure 3:**
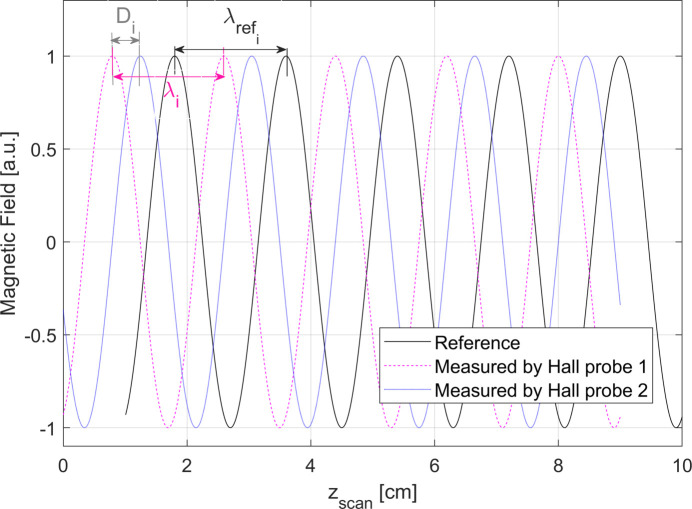
*Reference* sinusoidal magnetic field and magnetic fields *measured* by the first and second Hall probes as a function of the longitudinal position read by the encoder of the linear motion system.

Our setup is defined by an undulator where the λ_*i*_ value has been measured for the *i*th period length. Two Hall probes, placed at a fixed distance on the same sledge, are used for scanning simultaneously the magnetic field profile of the undulator. Fig. 3 ▸ illustrates the signal read by the two probes. If one Hall sensor experiences a displacement of *d*_HP_, the rod would have moved the nearest distance between adjacent peaks of the two Hall probes, *i.e.* *D*, shown in Fig. 3 ▸, which can be measured from the data. It means that

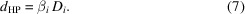

Since *d*_HP_ is known and *D*_*i*_ can be measured, we use them to estimate β_*i*_.

**Figure 4:**
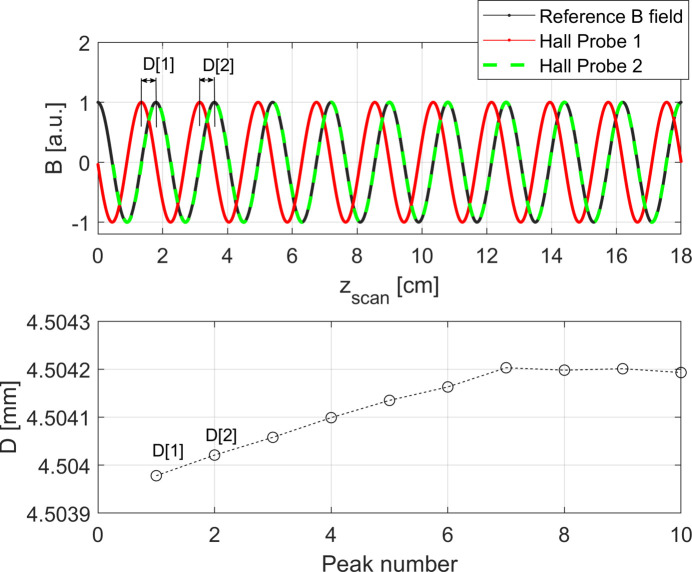
Top: example of magnetic fields *measured* by the two Hall probes compared with the *reference* one. Bottom: numerical array *D*. The calculation of the first two components of the array from the magnetic field recorded is schematically represented.

The corrected period lengths 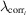 can then be calculated by using the equation 

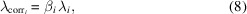

where 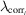 is the length of the *i*th period of the coil after the correction, λ_*i*_ is the measured value of the length of the *i*th period of the coil and *D*_*i*_ is the *i*th distance among the peak signals of the two Hall probes as illustrated in Fig. 4 ▸.

If Δ*L*_tot_ is strongly non-linear, this correction procedure can be improved by considering a higher order in the expansion of equation (5). The implication of this choice is that we will need to also increase the number of the Hall probes used for the measurement.

Let us consider for example the case in which we include the second-order term of equation (5). Following the same reasoning that we used for the linear case, it is possible to write 

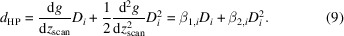

In order to measure β_1,*i*_ and β_2,*i*_, more Hall probes are required. Using three Hall probes it is possible to measure those quantities by solving the linear systems 

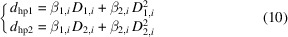

where *D*_1,*i*_ is the measured distance between the peaks of the first and second Hall probes at each period *i*, *D*_2,*i*_ is the measured distance between the peaks of the first and third Hall probe at each period *i*, *d*_hp1_ is the known distance in cold conditions between the first and the second Hall probes, and *d*_hp2_ is the known distance in cold conditions between the first and the third Hall probes.

## 3. Results

The goal of this section is to demonstrate the validity of the proposed method by applying it to simulations of magnetic field measurements with different experimental conditions. The study cases have been grouped according to the physical behaviour of the contraction of the rod during the scan of the Hall probe. Let us start by considering physical situations in which the contraction of the rod, Δ*L*_tot_, varies linearly with the position of the Hall probe read by the encoder, *z*_scan_.

### 3.1. Case studies with linear behaviour of contraction of the rod during scan of the Hall probe

#### 3.1.1. Linear temperature profile along the rod

**Figure 5:**
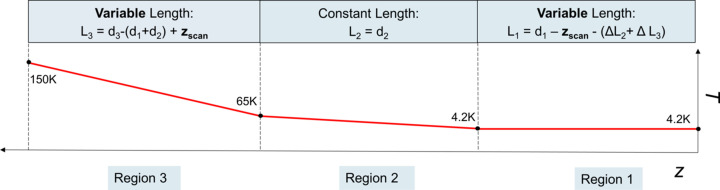
Conceptual sketch describing how the function *T*(*z*) is calculated at each point of the Hall probe scan *z*_scan_. 65 K and 150 K are the temperatures defined in Fig. 1 ▸. The variable *z* represents the longitudinal coordinate along the rod. *d*_1_ = 2.4 m is the maximum length of region 1 as defined in Fig. 1 ▸, *d*_2_ = 0.8 m is the fixed length of region 2, *d*_3_ = 4.5 m is the total length of the rod.

Our analysis begins with a physical case in which the dependence of *T* *versus* *z* in the three regions of the scan is linear, as depicted in Fig. 5 ▸. In the plot we can see the four points at the boundaries between different regions, where the temperature is fixed to 4.2 K, 4.2 K, 65 K and 150 K. The lengths *L*_1_ and *L*_3_ are variable and depend on *z*_scan_.

**Figure 6:**
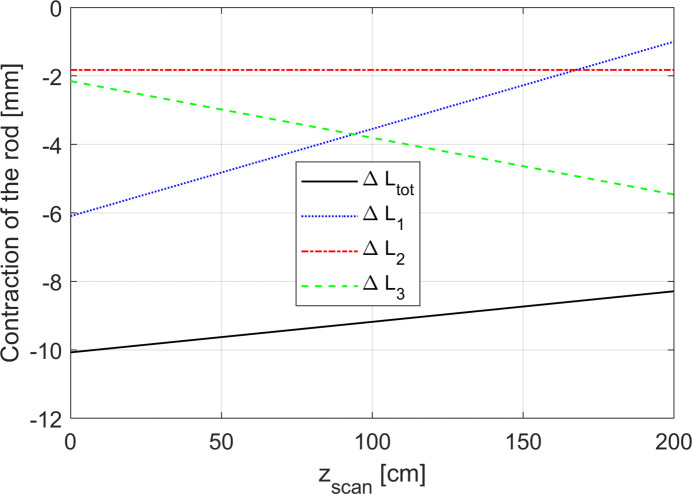
Behaviour of the total contraction of the rod Δ*L*_tot_ as a function of *z*_scan_. Δ*L*_1_, Δ*L*_2_, Δ*L*_3_ represent the contribution of the contraction/elongation of the rod in each region (1, 2, 3) described in Fig. 5 ▸ to its total contraction. It can be observed that Δ*L*_tot_ is linear in *z*_scan_.

The contraction of the length of the rod in each region as a function of *z*_scan_ is plotted in Fig. 6 ▸. The linear behaviour of the total contraction of the rod Δ*L*_tot_ is also represented. As can be observed, the rod is always contracted with respect to room temperature (Δ*L*_tot_ < 0) but its contraction decreases linearly, *i.e.* the rod expands linearly, during the scan. This observation matches our expectations since, during the Hall probe scan, the rod moves towards the warmer part of the cryostat, *i.e.* the top part.

It is also possible to note that the contribution (absolute value) of Δ*L*_1_ decreases, since region 1 becomes shorter for increasing *z*_scan_ (the rod moves out from the helium bath), while the opposite is true for region 3. As expected, the contribution of region 2 is constant during the scan.

**Figure 7:**
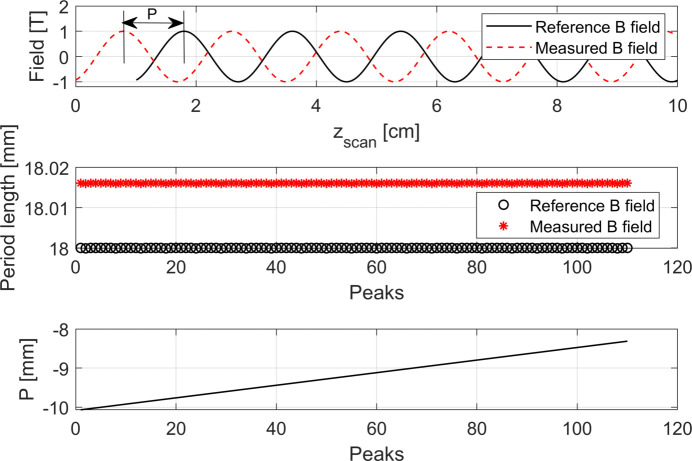
Upper plot: reference sinusoidal magnetic field and measured magnetic field as a function of the longitudinal position read by the encoder of the linear motion system scanning the longitudinal position of the Hall probe along the magnet. In order to be able to visualize the curves, only the first 10 cm of the 2 m scan-length are plotted. Middle plot: reference period lengths and measured period lengths that can be retrieved by analysing the two curves shown in the upper plot. Lower plot: distance between the peaks of the sinusoidal curve of the reference magnetic field and the ones expected from the measurement.

Our next step is to simulate a scan of the magnetic field of the coil. In the upper plot of Fig. 7 ▸ the curve of the magnetic field expected to be recorded in the Hall probe scan is presented:

(i) The *reference* magnetic field profile is given as 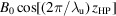 , with *z*_HP_ = *z*_scan_ − Δ*L*_tot_(*z*_scan_). In the latter expression, Δ*L*_tot_(*z*_scan_) is a function of *z*_scan_ (as evident from Fig. 6 ▸).

(ii) The *measured* magnetic field profile is obtained by sampling the reference field profile at *z*_HP_ but assigning to these sampled values the wrong coordinate *z*_scan_ as read by the encoder.

In the lowest plot in Fig. 7 ▸ it can be seen that the distance between the respective peaks among the reference magnetic profile and the one where we have not corrected the read-back of the encoder grows linearly with *z*_scan_. Such behaviour is due to the term Δ*L*_tot_ shown in Fig. 6 ▸. This results in a constant systematic error λ_err,C_ ≃ 16 µm, in the retrieval of the period lengths along the Hall probe scan in the coil, that is shown in the middle plot of Fig. 7 ▸.

**Figure 8:**
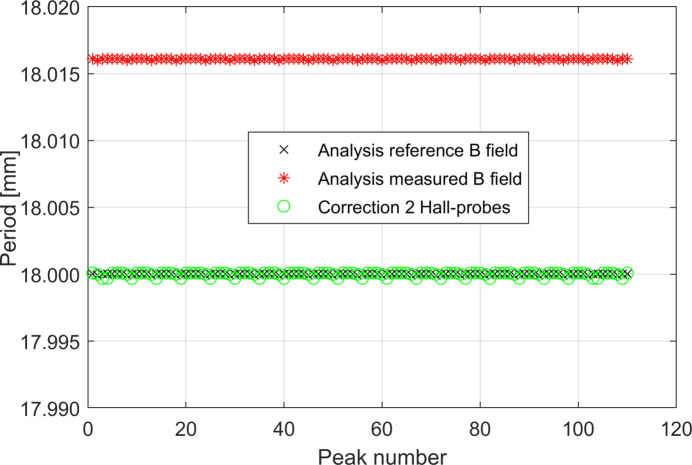
Effect of the correction procedure on the study case with the linear temperature function along the rod presented in Section 3.1.1.

Our next step is to try to neutralize this error by applying our correction procedure (Fig. 8 ▸). If the distance among the two Hall probes, *d*_HP_, which in our simulations is equal to 4.5 cm, *i.e.* one-quarter of the undulator wavelength, is known, the error of the measurement of the absolute value of the periods can be fully corrected.

The effectiveness of the correction of such a constant shift between the real and measured data is very sensitive to the accuracy to which the distance between the two Hall probes in cold conditions, *d*_HP_, is known. It is possible to derive analytically the expression for the systematic error λ_err,C_.

Recalling the definition (8) and omitting the indexes for the sake of readability, we can write

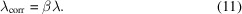

We can express the relative errors as 

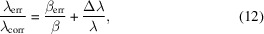

where Δλ is the statistic error on the measured period value λ and β_err_ is the error on the calculation of β. Using equation (11) combined with equation (12) we obtain 

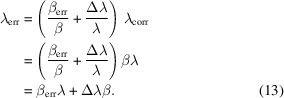

Recalling equation (7), we can express β = *d*_HP_/*D* and calculate the corresponding error propagation,

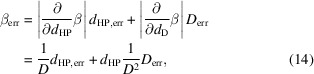

where *d*_HP,err_ is the error in the measured distance between the Hall probes and *D*_err_ is the statistical error in the measurement of *D*. Substituting equation (14) inside equation (13) we obtain

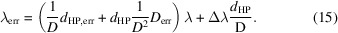

The error λ_err_ includes statistical errors connected to the measurement of the different parameters. It is interesting to extrapolate the sole contribution of the systematic error due to the positioning of the Hall probes by assuming that the statistical errors *D*_err_ and Δλ are equal to 0. We obtain 

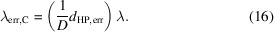

The behaviour described in this equation is in agreement with what we have observed in our simulations, where a binning for the magnetic field profiles of 0.1 µm has been used. This would correspond to a step of the Hall probe sensor of the same value (we will comment on the feasibility in Sections 4 and 5). In order to increase the accuracy for the determination of *D*, the data were fitted locally with a spline with binning 1 × 10^−9^. For this reason we can safely assume that *D*_err_ ≃ 0 and Δλ ≃ 0.

Equation (16) shows that by increasing the distance between the Hall probes it is possible to increase *D* and therefore decrease λ_err,C_. In real experiments the maximum distance between the Hall probes will be limited to a few centimetres or less, depending on the details of the setup, by mechanical requirements on the compactness of the sledge and rod.

#### 3.1.2. Uncompensated evaporation-rate of the helium in the cryostat

The case study analysed in the previous paragraph can be extended by adding also a variation of the helium level due to a constant heat load that is not compensated by any refill of the helium. We will see that, if the Hall probe travels with a constant velocity, the addition of this effect does not introduce non-linearity in the behaviour of Δ*L*_tot_ *versus* *z*_scan_.

In this new model, the temperature profile is composed of linear functions but also the central region of the rod, region 2, has variable length. Contrary to the model used in the previous section, the length of region 2 and the neighbour region 3 is a function of the position of the liquid helium evaporation surface, which is described by the function *z*_LiHe_(*z*_scan_). For this study we have used a more compact geometry of the cryostat with maximum length of region 1, *d*_1_ = 0.5 m; baseline length of region 2, *d*_2_ = 0.5 m; and total length of the rod, *d*_3_ = 2 m.

For simplicity we assume that the Hall probe moves with a constant velocity *v*_p_. We define 

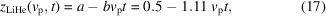

where the scaling factor *b* and constant *a* defining the variation of the liquid helium are selected arbitrarily. By using *z*_scan_ = *v*_p_ *t*, we can express the evaporation of the liquid helium directly as a function of *z*_scan_, 

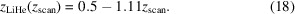

The function *z*_LiHe_(*z*_scan_) is linear and the helium levels vary around 0.5 m during the total duration of the scan.

**Figure 9:**
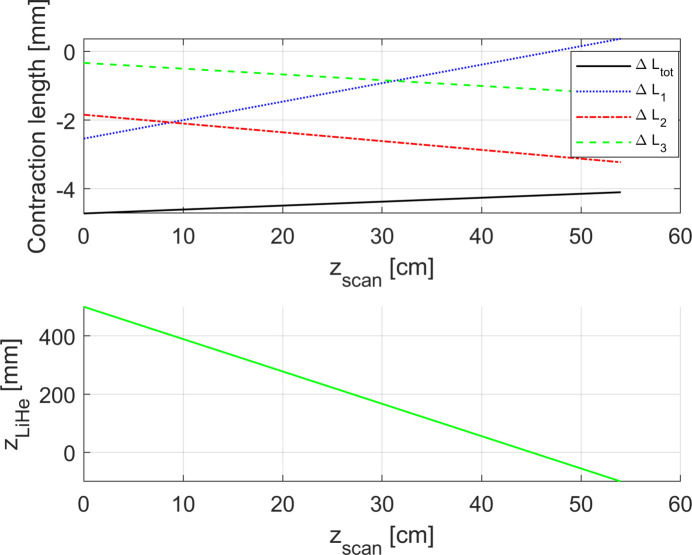
Upper plot: behaviour of the total contraction of the rod Δ*L*_tot_ as a function of *z*_scan_ for the simulated case with the linear temperature profile along the rod and uncompensated evaporation of the helium. Δ*L*_1_, Δ*L*_2_, Δ*L*_3_ represent the contribution of the contraction/elongation of the rod in each region (1, 2, 3). Lower plot: evolution of the liquid helium level as a function of the Hall probe position.

Fig. 9 ▸ presents the evolution of the total contraction of the rod Δ*L*_tot_ during the scan of the Hall probe. It can be seen that Δ*L*_tot_ is again a linear function of *z*_scan_.

**Figure 10:**
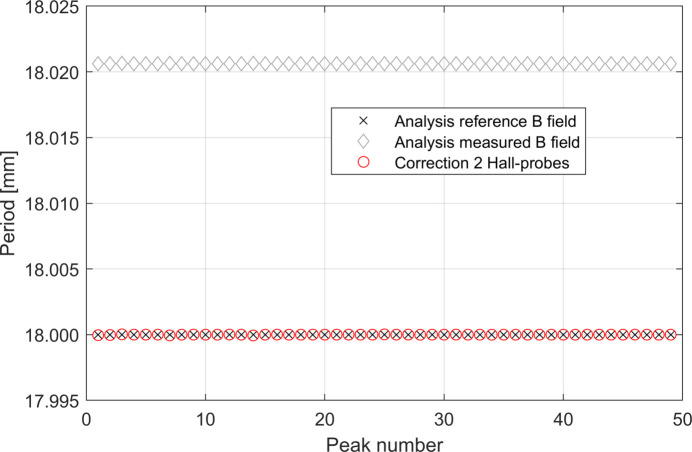
Calculated period lengths along the undulator by using the reference magnetic field and the measured magnetic field for the scan of Fig. 9 ▸. The offset λ_err,C_ between the reference and the measured values of the period length is about 20 µm. We also show the effect of the correction procedure. The latter curve is obtained considering *d*_HP,err_ = 0 µm.

Fig. 10 ▸ shows the systematic error on the measurement of the period of the undulator. As for the case presented in the previous section, when Δ*L*_tot_ is a linear function of *z*_scan_ we observe a constant systematic error λ_err,C_ on the measurement of the period length. We have applied our correction procedure also to this example; the results obtained in this case are plotted in Fig. 10 ▸. The same observations made in the previous example remain valid.

### 3.2. Case studies with non-linear contraction of the rod during the scan

This section is devoted to the presentation of physical case studies that can produce a non-linear behaviour of Δ*L*_tot_ *versus* *z*_scan_. Such non-linear behaviour can be generated in different ways. Here we will cover the cases where an exponential temperature distribution along the rod and a refill of the helium during the scan is present.

#### 3.2.1. Exponential temperature profile along the rod

**Figure 11:**
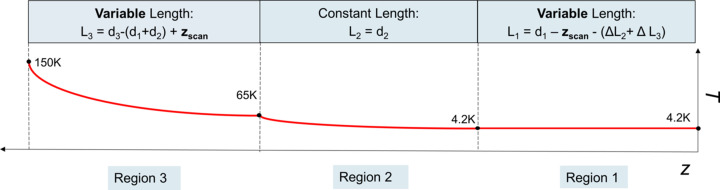
Conceptual sketch describing how the function *T*(*z*) is calculated at each point of the Hall probe scan *z*_scan_. *d*_1_ = 2.4 m is the maximum length of region 1 as defined in Fig. 1 ▸, *d*_2_ = 0.8 m is the fixed length of region 2, *d*_3_ = 4.5 m is the total length of the rod.

In experiments, it is expected that the temperature variation along the rod has a complex behaviour. Exponential fitting curves are, for example, used to fit real experimental data (Glasgow, 2009 ▸). For this reason our routines have been extended to model a temperature profile along the cryostat as the one depicted schematically in Fig. 11 ▸.

In this model the function *T*(*z*) is calculated by imposing an exponential fit in each region of the cryostat to match the fixed temperature boundaries among them. We want to calculate analytically the exponential curve *T*(*z*) = *a*exp(*z*) + *b* passing through the fixed points (*z*_*A*_, *T*_*A*_) and (*z*_*B*_,*T*_*B*_). Such a curve can be obtained by imposing

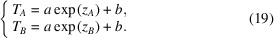

The solution of equations above is given by

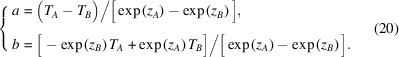

Such equations have been used to calculate the function *T*(*z*) in the three regions depicted in Fig. 11 ▸ at each position of the scan *z*_scan_.

**Figure 12:**
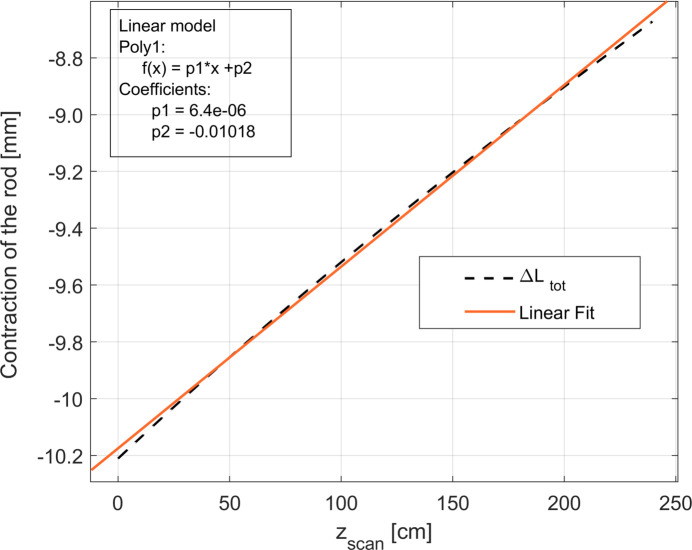
Behaviour of the total contraction of the rod Δ*L*_tot_ as a function of *z*_scan_. As the reader can see, the data do not fully overlap with the linear fit due to the presence of nonlinear terms.

Fig. 12 ▸ shows the total contraction of the rod Δ*L*_tot_ as a function of the position of the Hall probe. As can be graphically seen by comparing such a curve to a linear fit, in this case a mildly non-linear behaviour of the rod contraction during the scan is present.

**Figure 13:**
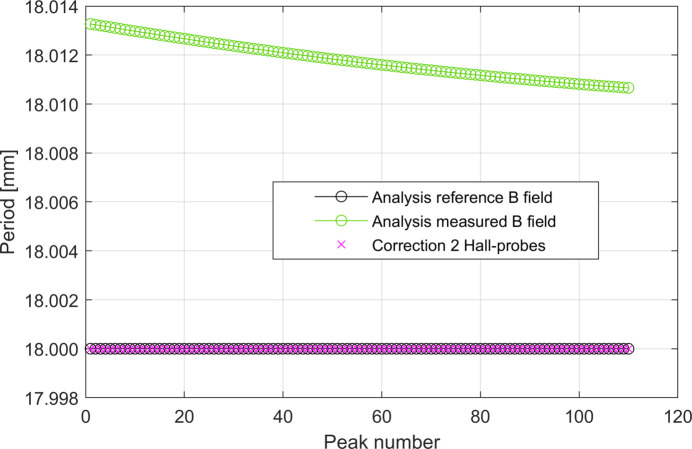
Calculated period lengths along the undulator by using the *reference* magnetic field and the *measured* magnetic field. The effect of the correction procedure is also shown. The latter curve is obtained considering *d*_HP,err_ = 0 µm.

Contrary to what has been found using the linear temperature function model, in this case the systematic error due to the contraction of the rod is variable along the scan, as shown in Fig. 13 ▸, and can be described by a discrete function λ_err_(*p*) = λ_err,C_ + *F*(*p*), where *p* = 1, 2,…, *N* indicates the consecutive number of the *N* peaks measured by the Hall probe. The use of two Hall probes proved to be good enough for correcting the systematic errors presented here.

**Figure 14:**
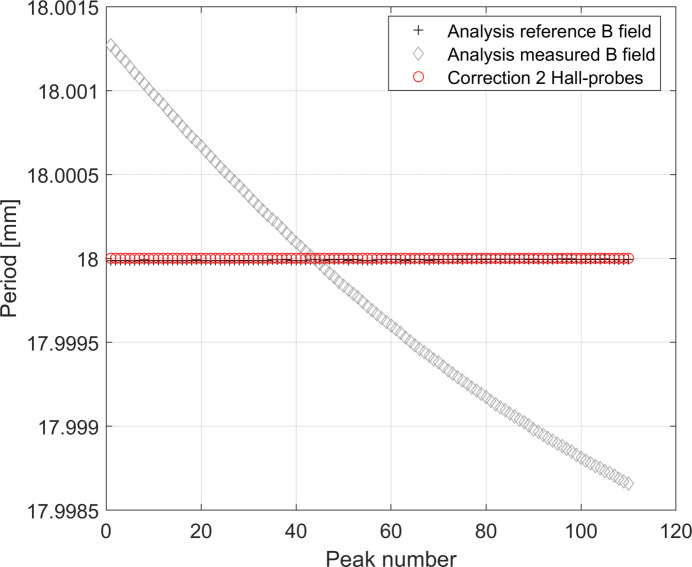
Effect of the correction procedure on the study case with exponential temperature function along the rod and considering *d*_HP,err_ = 300 µm and *d*_HP_ = λ_ref_/2. In the plot the red curve has been shifted by λ_err,C_ = 4*d*_HP,err_ and the grey curve has been shifted by 0.012 mm for a better visualization.

By looking at Fig. 14 ▸ it is interesting to note that, while a precision better than 1 µm in the measurement of *d*_HP_ is needed to correct λ_err,C_, a precision of 300 µm in the measurement of *d*_HP_ is sufficient to correct *F*(*p*).

This result is very relevant, since 300 µm is a realistic tolerance in the measurement of *d*_HP_.

#### 3.2.2. Periodical or one-time refill of the helium in the cryostat during the scan

In this paragraph the same physical model as described in Section 3.1.2 will be used but more complex functions for the description of the variation of the helium level will be applied.

(i) In our first study case we define 

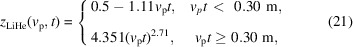

which again, assuming a constant velocity of the Hall probe, can be re-written as 

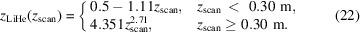

**Figure 15:**
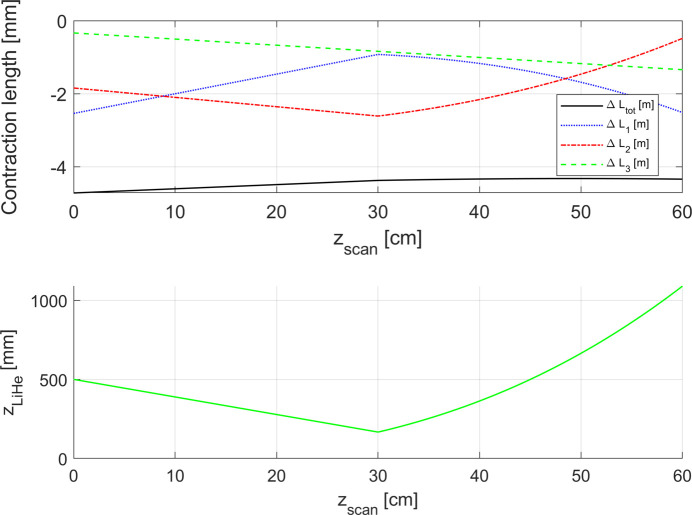
Upper plot: behaviour of the total contraction of the rod Δ*L*_tot_ as a function of *z*_scan_ for the first study case. Δ*L*_1_, Δ*L*_2_, Δ*L*_3_ represent the contribution of the contraction/elongation of the rod in each region (1, 2, 3). Lower plot: evolution of the liquid helium level as a function of the Hall probe position.

As can seen in Fig. 15 ▸, this function represents a simulated measurement during which we experience a constant evaporation of the liquid helium in the first part of the magnetic field scan, *i.e.* until *z*_scan_ = 0.30 m. Then the liquid helium is refilled in the cryostat and therefore the function *z*_LiHe_(*z*_scan_) increases non-linearly. The function *z*_LiHe_ (*z*_scan_) varies around 0.5 m during the total duration of the scan.

This example represents a malpractice case, since typically in experiments the helium is refilled either before or after the measurement.

(ii) In our second study case we define 

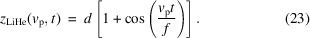

By choosing arbitrarily the parameters *d* and *f* and imposing constant velocity of the Hall probe, it is possible to rewrite the equation as 

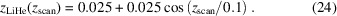

**Figure 16:**
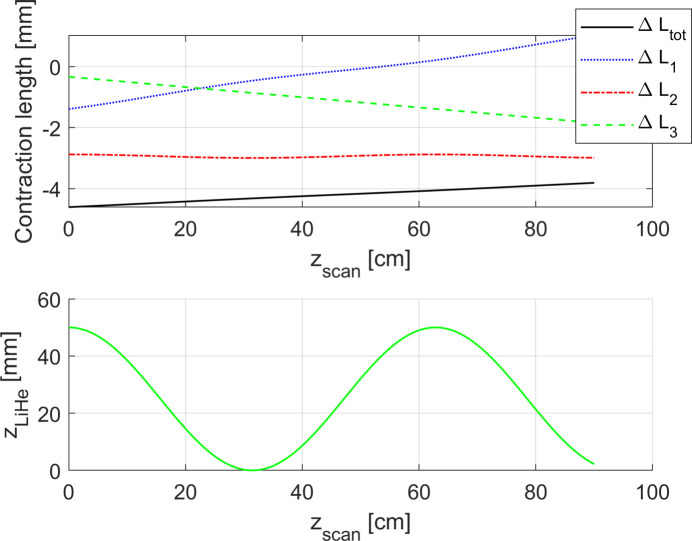
Upper plot: behaviour of the total contraction of the rod Δ*L*_tot_ as a function of *z*_scan_ for the second study case. Δ*L*_1_, Δ*L*_2_, Δ*L*_3_ represent the contribution of the contraction/elongation of the rod in each region (1, 2, 3). Lower plot: evolution of the liquid helium level as a function of the Hall probe position.

As visible in Fig. 16 ▸, this function represents a periodic oscillation of the liquid helium level in a range around 5 cm, which is comparable with the precision of the stabilization feedback in a typical cryostat.

Figs. 15 ▸ and 16 ▸ present the evolution of the total contraction of the rod Δ*L*_tot_ during the scan for the two study cases. It can be seen that in both cases Δ*L*_tot_ shows a non-linear behaviour.

**Figure 17:**
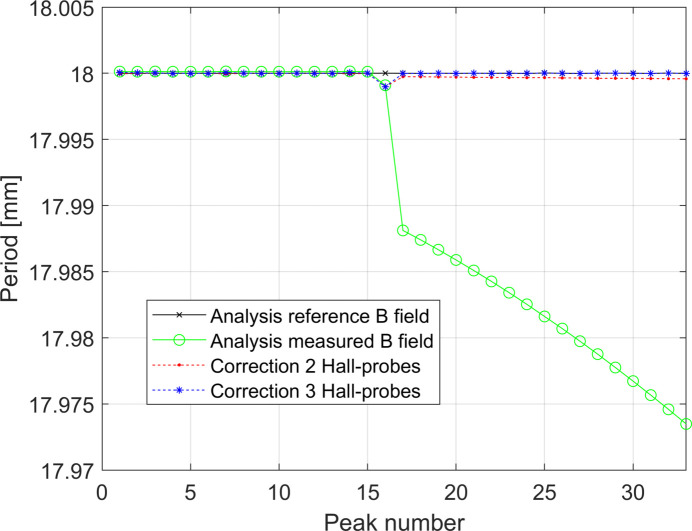
Periods of the undulator recovered by analysing the *reference* and *measured* magnetic fields of the simulation presented in Fig. 15 ▸. The *measured* magnetic field has been artificially shifted down by 20 µm for a better visualization of the component of the component *F*(*p*) of the systematic error. The refill of the helium during the measurement generates a systematic error starting from the 15th peak analysed. The corrected data are also plot. These curves are obtained considering *d*_HP,err_ = 0 µm.

Figs. 17 ▸ and 18 ▸ show the systematic error of the measurement of the period of the undulator in the two cases studied. As for the cases presented in the previous section, when Δ*L*_tot_ is not a linear function of *z*_scan_ a variable systematic error component *F*(*p*) in the region of the non-linearity of Δ*L*_tot_ is observed. As usual the correction procedure can be applied but it is worth noting that the contribution of *F*(*p*) for the latter study case is already of the order of 2 µm only.

**Figure 18:**
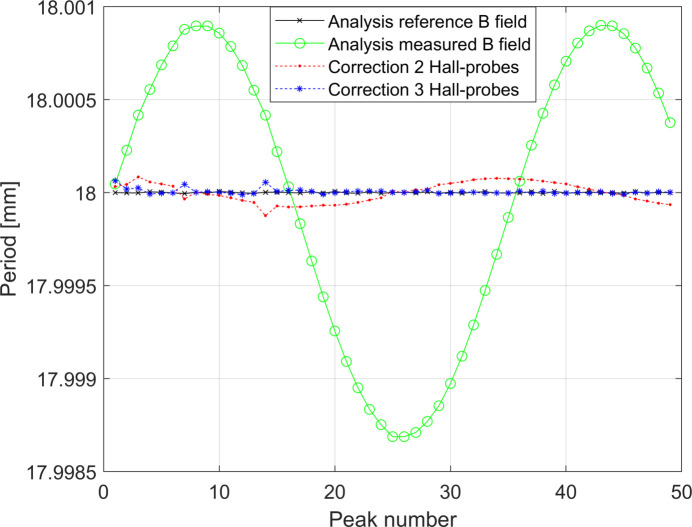
Periods of the undulator recovered by analysing the *reference* and *measured* magnetic fields for the simulation shown in Fig. 16 ▸. The *measured* magnetic field has been artificially shifted down by 15.9 µm for a better visualization of the component of the component *F*(*p*) of the systematic error. The periodic behaviour of the helium level during the measurement generates a periodic systematic error. The corrected data are also plot. These curves are obtained considering *d*_HP,err_ = 0 µm.

Although the correction using two Hall probes proved to be sufficient to correct the systematic errors below 1 µm, we have also tested the correction procedure supposing to have three Hall probes available. The obtained results, shown in Figs. 18 ▸ and 19 ▸, confirm that the addition of one Hall probe can correct further down the systematic error in the regions of the scan with strong non-linearity of Δ*L*_tot_. This observation confirms the validity of the analytical model that has been developed in this study.

## 4. Considerations on SUNDAE1

This section focuses on the application of the message learned in the study of SUNDAE1. For the experimental characterization of the magnetic field of the SCU coils to be used in the future afterburner of European XFEL, the position of the Hall probe in SUNDAE1 will be scanned with steps of 1 µm. Similar resolution and accuracy in the positioning of the Hall probe are requested for applications in storage rings. For the studies presented in Sections 3.1.1 and 3.2.1 we have considered geometrical dimensions of the cryostat similar to SUNDAE1. The simulations have shown that in the most general case when Δ*L*_tot_ is not a linear function of *z*_scan_ a variable systematic error λ_err_(*p*) = λ_err,C_ + *F*(*p*) in the measurement of the periods of the magnetic field is present. It is very important to distinguish this systematic measurement error from the real physical error in the manufacture of the coil. It is therefore critical from the experimental point of view to take into account such phenomena when analysing the data. A correction procedure involving the use of two Hall probes proved to be suitable to correct the systematic error in the measured period distribution. In the simulations presented, the distance between the Hall probe sensors was 4.5 mm; such a system is compact enough to be hosted on the sledge. The use of larger distances between the probes can further improve the robustness of the method with respect to the error in the positioning of the sensors. The simulations of the measurement have shown that, for the range of parameters which has been studied, an error of the order of 300 µm in the positioning of the Hall probes with respect to each other still allows the compensation of *F*(*p*) in the measurement below 1 µm.

The correction of the constant component of the systematic error λ_err,C_ could also be made in principle by analysing the signal from the two Hall probe sensors. However, to reach the goal resolution of 1 µm, which is requested for SUNDAE1, such a distance should be known with an accuracy of ≤1 µm using *d*_HP_ = 4.5 mm. In order to reduce λ_err,C_ below 1 µm the two Hall probes shall be put at a much larger distance (for example, *d*_HP_ = 18 cm with an accuracy in the positioning of 10 µm) that it is presently not realistic. For our purposes it is not interesting to measure the absolute value of the period lengths but rather their relative changes. This is due to the fact that the photon output in storage rings and FELs is influenced by relative deviations of the field and lengths in successive half-periods, while an offset on one of the two values can be compensated by properly tuning the current of the magnet. Moreover, possible correction schemes to deal with small but systematic errors of the machining of the magnet which could be detrimental for the phase error have been analysed and discussed by Grattoni & Casalbuoni (2023 ▸).

Finally in SUNDAE1 a cryogenic plant and a feedback system monitoring the helium level with a precision of a couple of cm will be available. We can therefore conclude that in such a setup the variation of the helium level should not compromise the accuracy of the magnetic field measurement.

As explained in Section 3.1.1, the simulations presented in this article did not consider errors in the calculation of the distance between the peaks of the magnetic fields measured by the Hall probes. Such errors will influence the accuracy of the corrected values of the periods as described in equation (15). For SUNDAE1, it has been checked that by increasing the sampling step of the Hall probe measurement to 1 µm (specified step for the linear motion system) we do not observe significant differences in the correction procedure.

## 5. Conclusions

This article focused on magnetic field characterization by using Hall probe measurements under cryogenic conditions. A general theoretical study aiming at the distinction between systematic thermal errors of the Hall probe measurement setup and real physical errors in the coil manufacturing has been performed. A novel correction procedure based on multiple Hall probe measurements has been derived and benchmarked with numerical simulations. The described correction procedure is generally applicable to vertical cryostats in which the Hall probe is permanently immersed in liquid helium and allows the correction of systematic errors caused by thermal contraction of the rod during the scan of the magnetic field.

We have verified that by applying the correction procedure to SUNDAE1 it is expected to be possible to achieve the goal performances for the analysis of the data.

Finally, it is important to recall the limitations of the presented study, that have been already mentioned during the course of the article.

First of all, in our numerical models we have considered a contraction coefficient for the rod α that is constant during the scan of the Hall probe position. It has been mentioned that this assumption is not true in reality, since α is a function of temperature. However it is important to notice that adding to our model the dependency α(*T*) will ultimately only provide a different shape for the specific function Δ*L*_tot_(*z*_scan_) corresponding to the experimental case under analysis. All the qualitative considerations regarding the expected characteristics of the systematic error and the procedure to correct it are still valid.

Secondly, we have simulated the measurement of an ideal undulator having periods with exactly the same length (18 mm). In reality the analysis will be applied to an undulator with a distribution of the period lengths which is centred around the value 18 mm.

As explained in Section 3.1.1, the simulations presented in this article did not consider errors in the calculation of the distance between the peaks of the magnetic fields measured by the Hall probes. Electronic errors of the Hall probes might still introduce visible effects on the result through this effect, influencing the accuracy of the corrected values of the periods as described in equation (15). Since the correction procedure that has been developed prescribes the measurement of the peaks of the magnetic field read by the two or more Hall probe sensors, we expect it to be not strongly affected by the calibration error. We leave the dissertation of the such effects to further future studies.

## Figures and Tables

**Figure 19:**
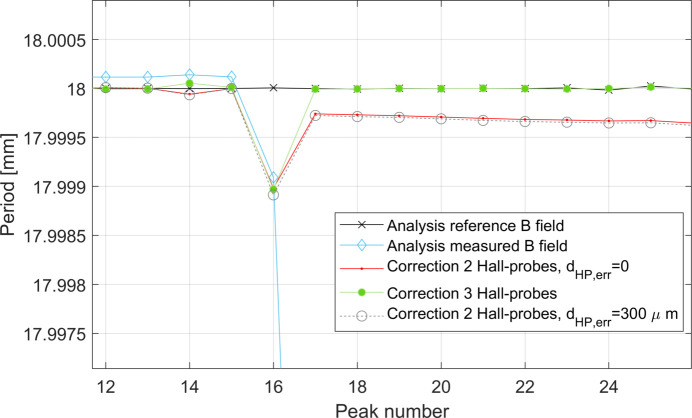
Effect of the correction procedure on the study case with helium refill during the measurement presented in Section 3.2.2. Here the plot presented in Fig. 17 ▸ has been zoomed and the curve obtained considering *d*_HP,err_ = 300 µm for the correction made using two Hall probes has been added. The offset of the different curves has been subtracted for a better visualization of the compensation of *F*(*p*).
